# Use of New Oral Anticoagulants / Direct Oral Anticoagulants in Malignant Patients

**DOI:** 10.7759/cureus.7007

**Published:** 2020-02-16

**Authors:** Yusra Khan, Syed Owais Zaidi, Bibi S Razak, Mariann Zaki, Bilal Haider Malik

**Affiliations:** 1 Pharmacy, California Institute of Behavioral Neurosciences and Psychology, Fairfield, USA; 2 Internal Medicine, California Institute of Behavioral Neurosciences and Psychology, Fairfield, USA; 3 Family Medicine, Nova Southeastern University School of Osteopathic Medicine, Sarasota, USA

**Keywords:** new oral anti-coagulants, atrial fibrillation in cancer patients, venousthromboembolisim in cancer patients, cancer assossiated thrombosis, rivaroxiban in cancer patients, apixaban in cancer patients, edoxaban in cancer patients, dabigatrin in cancer patients

## Abstract

Vitamin K antagonists are being used in the last five decades as an effective anticoagulant. However, for the past few years, new oral anticoagulants (NOACs) have been introduced as newer anticoagulant agents, which are gradually replacing the previously used vitamin K antagonist. Yet, these agents have not fully replaced the use of warfarin and heparin. NOACs have few advantages over the vitamin K antagonist as they act on a specific factor of coagulation cascade rather than inhibiting the whole vitamin K synthesis.

In this article, all the data has been searched electronically on PubMed and PRISMA guidelines were not followed. Instead, we used MOOSE statements and the data searched on PubMed was from articles published in the last five years.

A total of 12,269 patients were observed;,out of which 64.19% had active cancer and 35.80% was observed as a control group comprised of both male or female participants. Approximately 61.14% were using NOACs, 42.83% were on warfarin, and 2.72% were on low-molecular-weight heparin (LMWH). The NOACs used in different patients were in the following percentages; edoxaban (6.81%), apixaban (5.28%), dabigatran (10.09%), and rivaroxaban (10.02%).

The use of NOACs has been increasing day by day but these agents have not completely replaced the warfarin or heparin, because of some demerits associated with the use of warfarin and some conditions where these drugs should be avoided. All NOACs have either hepatic or renal clearance so the hepatic activity and creatinine clearance rate must be monitored before the start of NOACs. The drug interaction between anticancer drugs and NOACs is still not fully reported.

The effects of NOACs in AF and VTE are therapeutically effective, but in oncology patients several other co-factors are also involved with the use of NOACs due to which, it is either contraindicated or in some cases dose adjustment is required. However, very little information has been collected and more investigation must be done in this perspective.

## Introduction and background

According to Danny Hillis, “A human body is under continuous conversation, both within the cells and between the cells, and they coordinate with each other to grow and to die and when you are sick, something is gone wrong with that conversation”.

Warfarin and heparin are being used for more than 50 years as anticoagulants; yet, we need to carefully monitor the effect of VKA to avoid bleeding and loss of efficacy [1]. New oral anticoagulants (NOACs) have several benefits over the previously used anticoagulants [2].

The advancement of ximelagatran has set the basis of “NOACs”. In the year 2003, ximelagatran was accepted in Europe for short-term venous thromboembolism as a direct oral anticoagulant. This drug was rejected and removed from the market because of the rise in liver enzymes due to this drug, but this set the basis for the advancement of NOACs [1].

The difference between VKA and NOACs is that VKA works by reducing the hepatic synthesis of various clotting factors while NOACs work by acting on specific factors of the coagulation cascade [3]. Besides, NOACs do not require routine monitoring, while VKAs have a narrow therapeutic index, which can be very troublesome and costly for patients as well as healthcare providers. However, the biggest challenge of VKAs is to manage its interactions with food and other drugs [4].

Patients with malignancies are at higher risk of venous thromboembolism and arterial fibrillation. Deep vein thrombosis is a very common and life-threatening disease. According to a worldwide report, 20% of VTE is associated with cancer [5]. The risk of VTE and AF is 4 to 7 times greater in cancer patients. AF has affected roughly 30 million of the population and evidence suggests that AF and malignancies may coexist, which results in increased thromboembolism and bleeding complications and approximately 9,000,000 cases of VTE reported in the United States [6-7].

NOACs have several merits and demerits as compared to previously used anticoagulants. They have a wide therapeutic range and greater bioavailability, but there are risks of bleeding especially in cancer patients and several other risks associated with NOACs [2]. Today, NOACs are used equally as warfarin and some NOACs are considered superior to warfarin as they have low adverse effects as compared to warfarin, but still there are some major pitfalls in the use of NOAC drugs such as increased bleeding, elevation of liver enzymes, and others [8]. NOACs also have some challenging issues, as these are primarily excreted through the kidney, so the safety of these drugs in renal impairment is a great challenge [9-10].

In this article, we will be discussing the pharmacokinetics and pharmacodynamics of NOACs, their mode of action, their uses, and their safety and efficacy in cancer patients with possible drug interactions and risks associated with the use of NOACs in malignant patients such as the recurrence of thromboembolism [4]. This article also presents cost comparison of heparins and NOACs drugs, the identification of biomarkers of cancer-associated thrombosis, early treatment of thrombosis with NOACs, and the guidelines to use NOACs in cancer patients [11-12].

## Review

Method

This article is a traditional review article and the search was done electronically. 

Database

We used PubMed for our research analysis by using keywords.

Regular Keywords

We searched regular keywords and the results are mentioned in Table 1.

**Table 1:** Regular keyword

s.no	Keywords	Data base	Number of results
1	New oral anticoagulants	Pubmed	459
2	Atrial fibrilation in cancer patients	Pubmed	156
3	Venous tthromboembolism in cancer patients	Pubmed	435
4	Cancer-associated thrombosis	Pubmed	587
5	Rivaroxiban in cancer patients	Pubmed	41
6	Apixaban in cancer patients	Pubmed	21
7	Dabigatran in cancer patients	Pubmed	23
8	Edoxaban in cancer patients	Pubmed	15

MeSH Keywords

We searched MeSH keywords and the results are mentioned in Table 2.

**Table 2:** MeSH keywords

S. no	Keywords	Database	Number of results
1	Venous thrombosis	Pub med	2
2	Atrial fibrilliation	Pubmed	10
3	Anticoagulant drugs	Pubmed	12

Study Selection

Inclusion / Exclusion Criteria

This study used the following criteria: i) The patients must be using NOACs/DOACs. ii) Patients must have been detected for oncology. iii) All the patients are 18 years of age and above.

The data was collected from PubMed by using the filters of the past five years, free full text, and species human. All the literature used in the preparation of this article is derived from peer-reviewed articles, meta-analysis, systematic reviews, and some case studies. The literature from the year 2015 to 2019 is included. In this literature survey, we mainly focused on cancer patients using novel anticoagulant drugs, their effects, and pharmacology of drugs and our study was not based on a particular region.

Ethical Issue

All data was collected and used ethically and legally.

Quality Appraisal

In this literature survey, we used different assessment tools like AMSTAR CHECKLIST (systematic reviews, meta-analysis), qualitative reviews, clinical trials, Cochrane risk bias assessment tool, and JB Assessment tools (case reports).

Result

The search results on PubMed by the regular keywords for “New oral anticoagulants" were found to be 459: 156 for atrial fibrillation in cancer patients, 435 for venous thromboembolism in cancer patients, 587 for cancer-associated thrombosis, 41 for rivaroxaban in cancer patients, 21 for apixaban in cancer patients, 23 for dabigatran in cancer patients, and 15 for edoxaban in cancer patients. While with the MeSH keywords, the results were followed by venous thrombosis (2), atrial fibrillation (10), and anticoagulant drugs (12).

All the references used in this article are full-text articles from the last five years. Around 40 to 45 articles were shortlisted from PubMed for writing this review and all the articles were specified for cancer patients using NOACs as their anticoagulation therapy.

All these articles were in the English language. We did not need the language translator for these articles. Our study was not specific to a single region but mostly the research studies were U.K., Canada, and Europe based.

A total of 12,269 patients were observed;,out of which 64.19% had active cancer and 35.80% was observed as a control group comprised of both male or female participants. Around 61.14% were using NOACs, 42.83% were on warfarin, and 2.72% were on low-molecular-weight heparin (LMWH). A variety of NOACs such as edoxaban (6.81%), apixaban (5.28%), dabigatran (10.09%), and rivaroxaban (10.02%) were used in different patients. We did not use any quality assessment tools.

Discussion

Mechanism of Action of NOACs/DOACs

**Figure 1:**
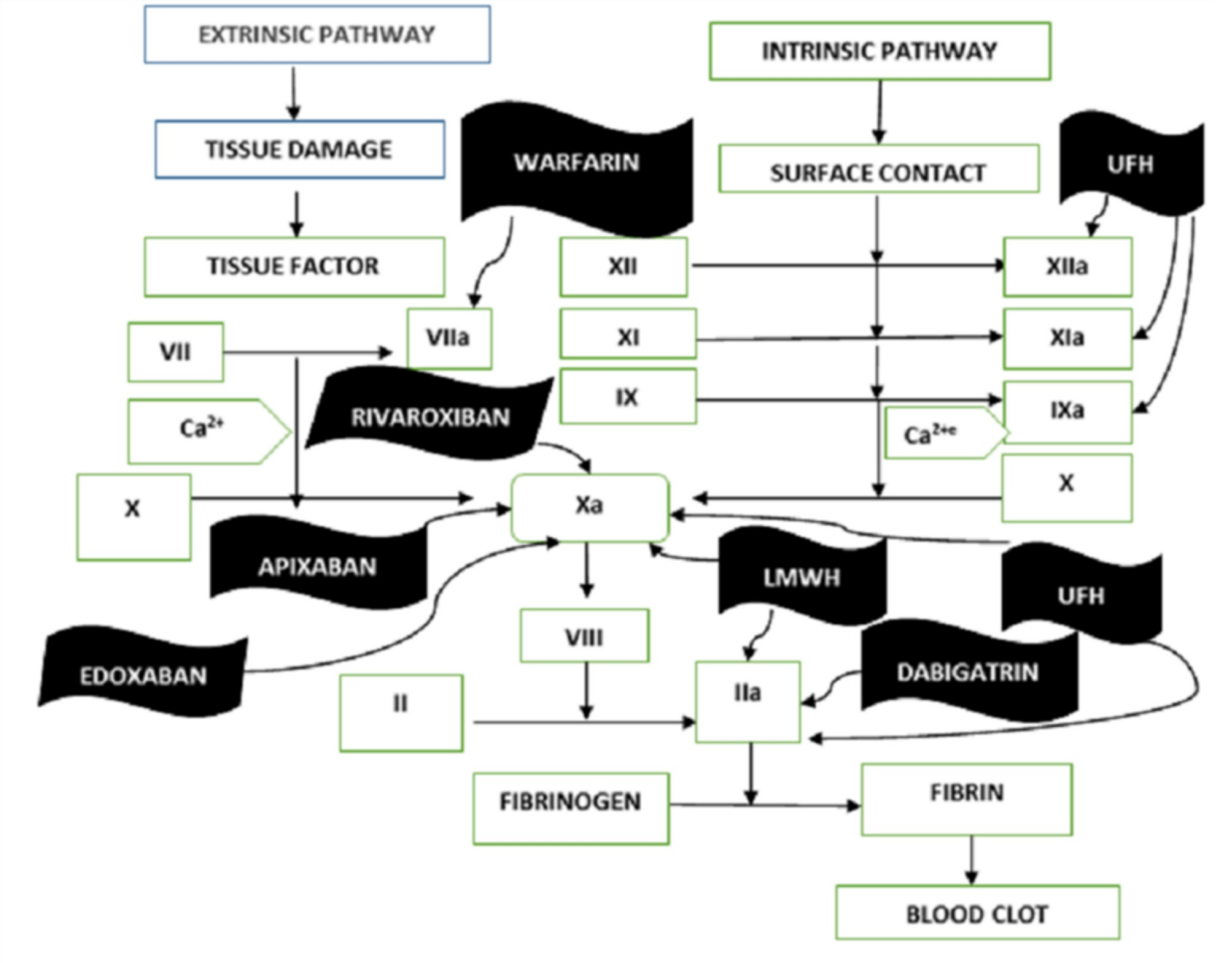
Mechanism of action LMWH: low-molecular-weight heparin; UF: unfractioned heparin

The coagulation of blood is a multistep process known as the coagulation cascade. This cascade formation is a systematic process of producing a new protein. This acts as a catalyst for both (extrinsic and intrinsic) pathways to produce a prothrombin activator. It triggers prothrombin conversion into thrombin, which is the final mediator in the fibrin formation. For more than 50 years, warfarin and heparin have been used to treat the coagulation in blood vessels. However, for the past few years the NOACs/DOACs have also been used. These drugs have not fully replaced warfarin and heparin but are surpassing it with their safety of use and a few other advantages over the previously used anticoagulants [1]. The mechanism of action of anticoagulants is represented in Figure 1.

One advantage of NOACs over warfarin is that they directly inhibit the coagulation protein by binding to their catalytic sides while the warfarin renders to inactivate the modification of vitamin K factors. Rivaroxaban, edoxaban, and apaxiban (NOACs) directly inhibit the factor Xa, while dabigatran inhibits thrombin directly. NOACs depend upon the CYP3A4 and P-gp for their metabolism [13]. Moreover, rivaroxaban and apixaban are dependent on CYT P450 3A4/3A5 for part of their metabolism. NOACs have a rapid onset of action. Dabigatran, edaxoban, rivaroxaban, and apixaban are p-glycoprotein substrate. These P-gps are present on the surface of the intestine, bile duct, and renal tubules, which play an efficient role in the transportation of xenobiotic outside the body [3,14]. Rivaroxaban reaches peak plasma concentration in 2-4 hours while the half-life of the drug is 15 hours. Rivaroxaban has 25% renal elimination. Dabigatran reaches plasma concentration in a half-hour and its half-life is 17 hours but is 80% eliminated through the kidney; however, it requires an enzyme esterase for its activation. Apixaban has a peak plasma concentration of 4 hours and a half-life of about 15 hours, while renal elimination is 25%. Edoxaban has a peak plasma concentration of about 1 hour and half-life is 11 hours. Renal excretion is about 39% [3-4,15-16].

Atrial Fibrillation and Cancer

Atrial fibrillation (AF) is an irregular or abnormal heart rate, which can result in serious implications. For example, an increased risk of having a stroke or a thrombosis. Nearly half of the AF population is associated with comorbid cancer. AF can be a risk marker in cancer patients [17]. AF found commonly in malignant patients because the incidence of AF and cancer increases with the increasing age of the patient [4]. AF and cancer often co-exist, which may result in thromboembolic events and bleeding complications in patients. It is a matter of fact that AF patients with malignancies have a higher risk of bleeding and thromboembolic complications than nonmalignant patients but patients with either active or remote cancer are at higher risk of AF complications, which may result in disequilibrium in the autonomous nervous system due to pain and stress [6, 18]. AF in cancer patients may be a comorbid state due to their older age, disbalance of electrolytes, hypoxia, and age-related metabolic disorders, thus the link between AF and cancer may be due to the above-determined factors [19]. The comorbidity profiles were quite similar to apixaban, dabigatran, and rivaroxaban for bleedings and strokes [20].

NOACs are effective alternatives to warfarin in AF patients but still, there is a lack of specific guidelines for the use of NOACs in patients with cancer and AF due to the limited data [6]. The NOACs including dabigatran, apixaban, rivaroxaban, and edaxoban have provided a non-inferior effect as compared to warfarin in stroke prevention [21]. The effect of NOACs is varied in different malignancies and their subtypes. In AF patients with cancer, the efficacy of NOACs is somewhat similar to that of warfarin. In comparing the drugs, we observed that apaxiban showed more potency as compared to warfarin in active cancer patients but not in remote cancer patients. Dabigatran and rivaroxaban showed the same risk of bleeding as warfarin. The choice of anticoagulant also varies with the stages of cancer [6].

Venous Thromboembolism and Cancer

A blood clot in a vein is known as venous thromboembolism (VTE). It is a very serious complication in cancer patients. The threat of VTE in malignant patients is 4 to 7 times higher than the non-cancer population [22]. VTE includes deep vein thrombosis and pulmonary embolism, which are great concerns for malignant patients; patients with high rates of cancer-associated thrombosis (CAT) are at a high risk of recurrent VTE and severe bleeding complications [23-25]. Their efficacy is the same as that of vitamin K antagonist; however, dabigatrin did not show a significant difference in bleeding complications while rivaroxaban and apixaban showed a significant difference in the bleeding complications of CAT patients [4]. Edoxaban has the same effect as that of LMWH [12]. NOACs have certain advantages but still, they have their challenges. The selection criteria of patients to use NOACs are quite challenging [26-27].

Use of NOACs in Different Cancers

Cancer associated with AF and VTE is a heterogeneous disease irrespective of its site and treatment [28]. The use of NOACs in different cancers depends upon the stages of cancer. The treatment of anticoagulation is quite complex in patients undergoing chemotherapies and can result in serious complications and adverse reactions [25]. The use of NOACs depends upon the risk and benefit ratio of the drugs. Some factors to be considered for prescribing NOACs in cancer patients receiving chemotherapies are; (i) investigation of drug-drug interaction, (ii) etiologies of thrombocytopenia, (iii) severity of cancer patients, (iv) risk of bleeding events, (v) elderly patients, (vi) renal impairment, (vii) interactions with CYP3A4 and p-glycoprotein, and (viii) medication history and a majority of other complications that need to be monitored [5,26,29]. The patients with active cancer in first-line therapy are considered for LMWH, but in some cases the parenteral drug cannot be used; still, there is very little evidence of the use of NOACs in cancer patients [4,22,30]. Many malignancies are managed by T-cell therapies [31]. Rivaroxaban in ambulatory malignant patients showed a lower rate of bleeding and thrombosis. The patients may be suffering from pancreatic cancer, breast cancer, genitourinary cancer, lung cancer, ovarian cancer, gastroesophageal cancer squamous cell carcinoma, adenocarcinoma, and lymphoma [28,32-33]. In ambulatory patients, rivaroxiban showed a decreased risk of VTE but major bleeding occurred in few cases and in some cases, the reoccurrence of VTE and bleeding increased [23,33]. In primary and metastatic brain cancer, this drug showed more safety than LMWH [34].

Apixaban showed decreased events of VTE but possesses higher bleeding events in the patients suffering from lung, colon, and pancreatic cancer and undergoing chemotherapies but thromboprophylaxis was an effective and safe treatment with NOACs [35]. It also suppresses the activity of fVIIa (increase activity of VEGF), which proved to be an agent for controlling the proliferation of cancer cells as well as the risk of thrombosis [36].

Edoxaban considered an alternative of anticoagulants in cancer patients except for non-melanoma skin cancer or hematological malignancies [22]. Dabigatran and other anticoagulants showed no difference in the efficacy in the treatment of cancer patients except for the squamous cell carcinoma [22].

Nearly all anticoagulants, especially NOACs, increase the risk of gastrointestinal (GI) bleeding by forming a lesion on the lumen of GI tract and LMWH is preferred over rivaroxaban for the treatment of urinary tract lesions and by observing the result of few cases, we found that the incidence of bleeding in lower gastrointestinal tract (GIT) is greater than upper GIT [34,37].

Still, the NOACs cannot be compared directly with LMWH in malignant patients but it is considered that these drugs will prove even better than the previously used LMWH [30]. In the use of NOACs, there is less occurrence of bleeding and mortality rate, yet the quality of life is compromised and leads to lifelong medication [38].

Bleeding

Bleeding risk in cancer patients is not a single factor complication and is always multifactorial [12]. This may include thrombocytopenia, intravascular coagulation, elaboration of fibrinolytic factors in cancer patients. Most commonly, bleeding occurs in patients with renal and gastrointestinal cancers tissue damage; chemotherapies of bone marrow suppression radiations post-surgical wound healings also contribute to a higher incidence of bleeding risk in malignant patients [12,17]. Although the bleeding risk among the use of NOACs is less than warfarin but still in some cases as explained above bleeding occurs also with the use of NOACs [21].

Reversal Agents

In cancer patients, the bleeding treatment is largely dependent on the severity of disease and may vary from one patient to another although NOACs are safer than warfarin because bleeding events are less frequent in cases in which severe bleeding reversal agents are used [21, 30].

The European Heart Rhythm Association Practical Guide classifies several reversal agents.

Idaruximab is a monoclonal antibody fragment and acts as an antagonist of dabigatran. Andexanet alpha is a recombinant modified human decoy protein that acts as an antagonist of all factor Xa inhibitors (apixaban, rivaroxaban, and edaxoban). Ciraparantag is the antagonist of edaxoban while the administration of four prothrombin factor (II, VII, IX, X) complex is a rapidly used reversal agent for all NOACs [21]. In the case of minor bleeding, other possible strategies were applied such as skipping a few doses, sometimes dose reduction is also beneficial [30].

Thrombocytopenia

Thrombocytopenia is the decrease in platelet count, which is normally 100 x10 9 /L and is common in malignant patients especially those who are undergoing chemotherapies and suffering from hematologic cancers [12]. It can also be a result of a tumor invasion bone marrow. If thrombocytopenia occurs in cancer patients, the risk of bleeding is increased if the platelet count is above 50 G/L then full doses of NOACs can be used but if the platelet count of a patient is reduced from 50 G/L, then the doses may be adjusted from patient to patient [27].

Renal Insufficiency

Today NOACs are emerging as an alternative to warfarin. However, there are still some challenging situations in the use of these agents as they have very predictable pharmacology and a short half-life as compared to warfarin. However, all the NOACs have partial renal elimination, which makes the use of this drug a challenge in the patients with unpredictable renal results and there is a greater risk of bleeding events. Still, there is a lack of data in chronic kidney disease and end-stage kidney disease, the doses, and selection of NOACs are adjusted in different patients by determining there creatinine clearance rate [9]. Dabigatran is 80% eliminated through kidneys in renal impaired patients and the half-life of the drug increases up to two folds. Notable reductions were observed in major bleeding in patients with eGfr up to 80 ml/min, but not in renal impaired patients. In end-stage renal disease (ESRD) patients, which were on hemodialysis, the removal of dabigatrin from circulation has proved effective. Edoxaban is 50% eliminated through kidneys, the dose reduction appeared to be effective. Rivaroxaban is 30% excreted through kidneys and there is a decrease in clearance that produces a predictable drug exposure. In hemodialysis, there is no reduction in the plasma concentration of the drug. Apixaban is excreted about 25% through kidneys and the reduced dose in the renal impaired patients was found to be effective as compared to all NOACs. Apixaban showed superiority over warfarin in the prevention of stroke, embolism, bleeding, and even mortality rate has decreased the pharmacokinetics of all NOACs, which is affected by the activity of p- glycoprotein and cytochrome P450 3A4 [10,39].

Hepatotoxicity

NOACs are also metabolized by the liver enzyme CYP450 3A and may sometimes produce symptoms which result in elevated enzymes in patients. This question arose because ximelagatran showed hepatotoxic symptoms in its initial stages while the currently used NOACs found no hepatic risk [1,8]. Although NOACs induced hepatotoxicity is unusual, it has evidence of drug-induced liver injury as well as elevated enzyme levels of the liver [8]. There is a little more evidence which suggested that NOACs cannot be used in hepatic impairment [27]. Further investigation is required in this field.

Drug-Drug Interaction

Drug-drug interaction is a major area of concern because the majority of chemotherapies interact with NOACs, but still there is a lack of information [12]. All NOACs are contraindicated with macrolides, verapamil, and naproxen, while especially rivaroxibanis contraindicated with azoles and protease inhibitors [2]. Although there is no organized data for the drug interactions of NOACs with chemotherapeutic agents based on their interaction with CYP3A4 and P-gp and this data is generated [19]. The drug-drug interaction is represented in Table 3.

**Table 3:** Drug–drug interaction

S NO	ANTI CANCER DRUGS	MECH OF ACTION	EFFECT OF NOACS/DOACS				CONC. OF NOACS IN PLASMA	REF
			DABIGATRIN	RIVAROXIBAN	APIXABAN	EDOXABAN		
ANTIMITOTIC AGENTS								
1	Paclitaxel	Moderate induction of CYP3A4	No relevant interaction	Avoid or used with care	Avoid or used with care	No relevant interaction	Decreased	[4] [14] [18]
2	Vincristine	Mild induction of CYP3A4	No relevant interaction	Caution or dose adjustment	Caution or dose adjustment	No relevant interaction	Increased	[4] [14] [18]
3	Vinblastine	Strong P-gp induction	May not be used	May not be used	May not be used	May not be used	Decreased	[4] [14] [12]][18]
4	Doretaxel	Mild CYP3A4 induction	No relevant interaction	Caution or dose adjustment	Caution or dose adjustment	No relevant interaction	Increased	[4] [14][18]
5	Vinorelibine	Mild CYP3A4 induction	No relevant interaction	Caution or dose adjustment	Caution or dose adjustment	No relevant interaction	Increased	[4] [14][18]
TOPOISOMERASE INHIBITORS								
6	Etoposide	Mild CYP3A4 inhibition	No relevant interaction	Caution or dose adjustment	Caution or dose adjustment	No relevant interaction	Increased	[4] [14] [18]
ANTHRACYCLINES								
7	Doxorubicin	Strong P-gp induction, mild CYP3A4 inhibition	May not be used	May not be used	May not be used	May not be used	Decreased	[4] [14]
8	Idarubicin	Mild CYP3A4 inhibition	No relevant interaction	Caution or dose adjustment	Caution or dose adjustment	No relevant interaction	Increased	[4] [14]
ALKYLATING AGENTS								
9	Ifosfamide	Mild CYP3A4 inhibtion	No relevant interaction	Caution or dose adjustment	Caution or dose adjustment	No relevant interaction	Increased	[4] [14] [18]
10	Cyclo phosphamide	Mild CYP3A4 inhibtion	No relevant interaction	Caution or dose adjustment	Caution or dose adjustment	No relevant interaction	Increased	[4][14] [18]
11	Lomustine	Mild CYP3A4 inhibtion	No relevant interaction	Caution or dose adjustment	Caution or dose adjustment	No relevant interaction	Increased	[4] [14] [18]
TYROSINE KINASE INHIBITOR								
12	Imatinib	Strong P-gp inhibition, moderate CYP3A4 inhibition	Not recommended	Not recomended	Not recomended	Not recommended	Increased	[4] [12] [14] [18]
13	Nilotinib	Moderate to strong P-gp inhibition, mild CYP3A4 inhibition	Caution or dose adjustment	Caution or dose adjustment	Caution or dose adjustment	Caution or dose adjustment	Increased	[4] [12] [14] [18]
14	Vemurafaneb	Moderate CYP3A4 induction	No relevant interaction	Avoid or used with care	Avoid or used with care	No relevant interaction	Decreased	[4] [14][18]
15	Dasatinib	Mild CYP3A4 inhibition	No relevant interaction	Caution or dose adjustment	Caution or dose adjustment	No relevant interaction	Increased	[4] [14] [18]
16	Vandetanib	Strong P-gp induction	May not be used	May not be used	May not be used	May not be used	Increased	[4] [14] [18]
HARMONAL AGENTS								
17	Abiraterone	Medium CYP3A4 inhibition, strong P-gp inhibition	Not recommended	Not recomended	Not recomended	Not recommended	Increased	[4] [14] [18]
18	Enzalutamide	Strong CYP3A4 induction, strong P-gp inhibition	Not recommended	Not recomended	Not recomended	Not recommended	Decreased	[4] [12] [14] [18]
19	Bicalutamide	Medium CYP3A4 inhibition	No relevant interaction	Caution or dose adjustment	Caution or dose adjustment	No relevant interaction	Increased	[4] [12] [14] [18]
20	Tamoxifen	Strong P-gp inhibition, mild CYP3A4 inhibition	Caution or dose adjustment	Caution or dose adjustment	Caution or dose adjustment	Caution or dose adjustment	Increased	[4] [12] [14] [18]
21	Anastrazole	Mild CYP3A4 inhibition	No relevant interaction	Caution or dose adjustment	Caution or dose adjustment	No relevant interaction	Increased	[4] [14] [18]
IMMUNE MODULATING AGENTS								
22	Cyclosporin	Strong to moderate P-gp inhibition, medium CYP3A4 inhibition	Not recommended	Caution or dose adjustment	Caution or dose adjustment	Dose adjustment	Increased	[4] [14] [18]
23	Dexamethasone	Strong induction of both CYP3A4 and P-gp	May not be used	May not be used	May not be used	May not be used	Decreased	[4] [12] [14] [18]
24	Tarcolimus	Strong to moderate P-gp inhibition, mild CYP3A4 inhibition	Not recommended	Dose adjustment	Dose adjustment	Dose adjustment	Increased	[4] [14] [18]
25	Prednisone	Medium CYP3A4 induction	No relevant interaction	Avoid or used with care	Avoid or used with care	No relevant interaction	Decreased	[4] [14] [18]
26	Temsirolimus	Mild CYP3A4 inhibition	No relevant interaction	Caution or dose adjustment	Caution or dose adjustment	No relevant interaction	Increased	[4] [14] [18]

Cost

The expenditure of both OACs and NOACs was compared in different healthcare systems and we observed the studies from three different countries: China, Australia, and the USA. The results showed that the total expenditure of NOACs was quite less than the previously used OACs [11, 40-41].

New on Horizon

A direct antineoplastic effect of NOACs provokes an interesting area of research that needs to be reinforced before concluding scientific efficacy [22]. The total quantity of cell re-infusion seems to influence the growth of thrombosis, which is related to blood viscosity, but the exact reasons need more advanced research in the future [31]. Still, the data collected thus far is very little and more information is needed. In addition, the use of NOACs must be prescribed on the practitioner's advice; still, there is very little data on drug-drug interaction between anticancer and NOACs [14,42].

Limitations

For this study, we selected oncology patients from the last five years who were using NOACs as their anticoagulant therapy. The age range was from 25 onwards and both male and female patients were considered for the study. We did not use any quality assessment tools, all articles were free, full-text articles, we did not purchase an article, all articles were in the English language and this study was based on the use of NOACs/DOACs in oncology patients in the age range from 25 to 85 years in males or females.

## Conclusions

Anticoagulants such as warfarin and heparin are being used for anticoagulation therapy for more than 50 years. No such drug in history has been used so long, but now the oral anticoagulants are nearly replacing the previously used vitamin K antagonist. There are certain benefits of using NOACS over warfarin and heparin as they do not require regular monitoring, besides their effect is predictable.

However, the NOACs cannot completely replace the warfarin or heparin because all NOACs have either hepatic or renal clearance so the hepatic activity and creatinine clearance rate must be monitored before the start of NOACs.

The use of NOACs in atrial fibrillation and venous thromboembolism has proven to be quite safe and the bleeding risk is also reduced. The use of NOACs in different cancers depends on several other co-factors; for example, in GI cancers, the risk of bleeding has increased the use of NOACs and may be contraindicated or recommended on dose adjustment.

The interaction between NOACs and anticancer drugs is not fully reported, the data on the drug-drug interaction of NOACs and anticancer agents are lacking. Some data is reported in the European guidelines, but still, further research needs to be done in this aspect. Today, however, the use of NOACs is given priority over warfarin as the NOACs are available at a reasonable cost as compared to warfarin and heparin.
